# Posttraumatic Growth, Positive Psychology, Perceived Spousal Support, and Psychological Complications in Head and Neck Cancer: Evaluating Their Association in a Longitudinal Study

**DOI:** 10.3389/fpsyg.2022.920691

**Published:** 2022-06-24

**Authors:** Nik Ruzyanei Nik Jaafar, Norhaliza Abd Hamid, Nur Amirah Hamdan, Rama Krsna Rajandram, Raynuha Mahadevan, Mohd Razif Mohamad Yunus, Hazli Zakaria, Noorsuzana Mohd Shariff, Rohayu Hami, Salbiah Isa, Nurul Izzah Shari, Mohammad Farris Iman Leong Bin Abdullah

**Affiliations:** ^1^Department of Psychiatry, Universiti Kebangsaan Malaysia Medical Centre, Kuala Lumpur, Malaysia; ^2^Department of Community Health, Advance Medical and Dental Institute, Universiti Sains Malaysia, Kepala Batas, Malaysia; ^3^Department of Oral and Maxillofacial Surgery, Universiti Kebangsaan Malaysia Medical Centre, Kuala Lumpur, Malaysia; ^4^Department of Otorhinolaryngology, Universiti Kebangsaan Malaysia Medical Centre, Kuala Lumpur, Malaysia; ^5^School of Human Resource Development and Psychology, Faculty of Social Sciences and Humanities, Universiti Teknologi Malaysia, Skudai, Malaysia

**Keywords:** posttraumatic growth, head and neck cancer, positive psychology, perceived spousal support, depression, anxiety

## Abstract

Despite head and neck cancer (HNC) association with various negative impacts, collective evidence is accumulating regarding the positive impacts of positive psychology on cancer survivors. However, data on how positive psychology is related to the psychological complications of HNC across time are lacking. This longitudinal study examined the trends of positive psychology (e.g., posttraumatic growth [PTG], hope, and optimism), perceived spousal support, and psychological complications (e.g., depression, anxiety, and posttraumatic stress symptoms) and determined the association between them, psychological complications, and PTG across two timelines among a cohort of HNC patients. A total of 175 HNC respondents exhibited an increasing trend of positive psychology and perceived spousal support while reporting a decreasing trend of psychological complications between baseline and follow-up assessments. A greater degree of hope and perceived spousal support contributed to a higher degree of PTG across time. Conversely, a higher severity of anxiety symptoms was associated with a lower degree of PTG over time. Female gender had a moderating effect on the association between severity of anxiety symptoms and PTG, but did not moderate the association between hope, perceived spousal support and PTG. This study indicates the pivotal role of incorporating psychosocial interventions into the treatment regimen to enhance the degree of hope and perceived spousal support and reduce the severity of anxiety symptoms, which, in turn, will facilitate the development of PTG in HNC patients.

## Introduction

Head and neck cancer (HNC) refers to malignant tumors that develop around the head and neck area, such as the throat, mouth, nose, and ear (National Cancer Institute, 2021). The worldwide incidence of HNC is ~900,000 cases, whereas its mortality rate is as high as 400,000 deaths annually (Global Cancer Observatory, 2021). HNC contributes to a wide range of psychosocial issues, such as depression, anxiety, substance use disorder, suicide, conflict in interpersonal relationships, damage to self-esteem, social isolation, and facial disfigurement (Smith et al., 2017). It must be properly managed to ensure positive outcomes in the physical and mental wellbeing of HNC patients.

Notwithstanding these negative impacts of HNC, positive psychology, such as posttraumatic growth (PTG), hope, and optimism, has been reported in HNC patients (Ho et al., 2011; Leong Abdullah et al., 2015). PTG is defined as a positive psychological change resulting from struggles with highly challenging life crises or traumatic events. A greater degree of PTG in cancer patients may result in a better appreciation of life, improved interpersonal relationships, greater spiritual development, more possibilities in life, and enhancement of the personal strengths of patients (Calhoun and Tedeschi, 2006). PTG develop when cognitive processing and reappraisal of the traumatic event of living with cancer occurred. When there is integration of the trauma-related information to facilitate the reconstruction of the new assumptive worldview on self, others and the surrounding environment, *via* the process of accommodation, search of meaning out of the trauma of living with cancer occurred. A successful search of meaning to solve unproductive ruminations such as cognitive posttraumatic stress symptoms and fear of cancer progression, and facilitate the reconstruction of the new assumptive worldview on self, others and the surrounding environment, will lead to development of PTG (Leong Abdullah et al., 2019; Ochoa Arnedo et al., 2019).

**Figure 1:**
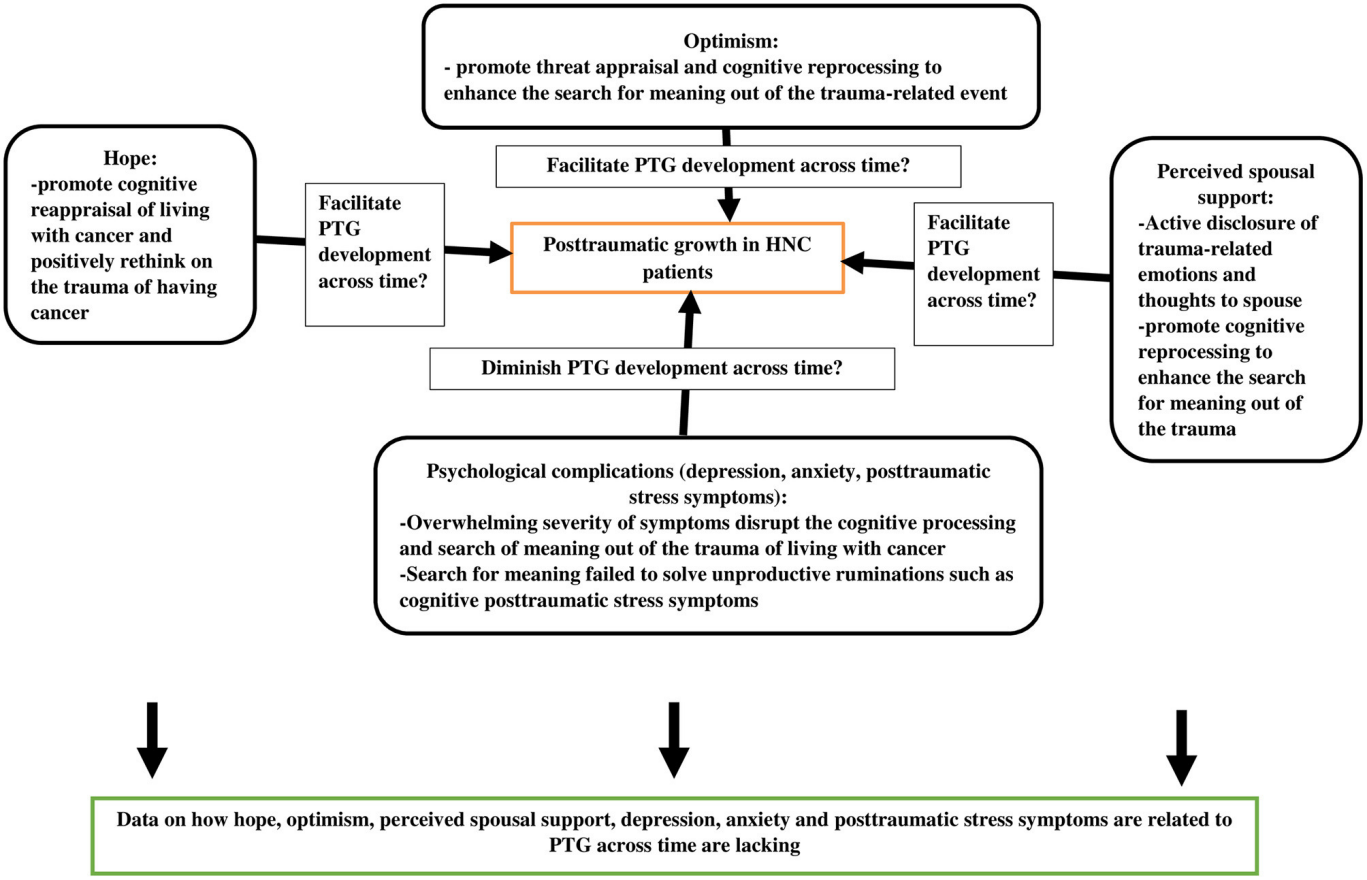
The theoretical framework of the possible association between hope, optimism, perceived spousal support, psychological complications related to cancer and posttraumatic growth among cancer patients.

The relationship between hope, optimism, and PTG in cancer patients remains inconsistent; some studies have revealed a direct association, whereas others have reported no association between them (Shand et al., 2015; Casellas-Grau et al., 2017). In the context of HNC patients, Ho et al. (2011) conducted the only cross-sectional study that investigated the relationship between hope, optimism, and PTG. The study reported that only the pathway domain of hope was associated with PTG and not the agency domain of hope and optimism (Ho et al., 2011). Hope may promote cognitive reappraisal of living with cancer and positively rethink on the trauma of having cancer. Hope also facilitates the acceptance of the traumatic event and leads to reconstruction of the of the new assumptive worldview on self, others and the surrounding environment. Hence, it may contribute to higher PTG among cancer patients (Leong Abdullah et al., 2019). In the context of optimism, Tedeschi and Calhoun (2004) stated that people with higher degree of optimism tend to concentrate on the most important points in life and forego unachievable goals and worldviews that are no longer consistent with the trauma-related event of living with cancer, which in turn will facilitate threat appraisal and cognitive reprocessing enhancing the search for meaning out of the trauma-related event, hence this promotes the development of PTG among cancer patients. The theoretical framework of the possible association between hope, optimism, perceived spousal support, psychological complications related to cancer and PTG are illustrated in Figure 1. To date, no longitudinal study has focused on the assessment of the relationship between these forms of positive psychology in HNC patients.

Social support forms a bidirectional relationship with positive psychology in cancer patients. Specifically, spousal support helps promote the active disclosure of life events and crises of cancer patients to their partners; this encourages cognitive reappraisal of the traumatic event of cancer diagnosis, thus fostering meaning making out of the cancer experience and eventually leading to PTG (Tedeschi and Calhoun, 2004; Shand et al., 2015; Figure 1). In the context of Malaysian cancer patients, PTG was found to be positively associated with perceived spousal support, particularly instrumental support (Schroevers and Teo, 2008; Leong Abdullah et al., 2019). However, to date, no longitudinal study has investigated how perceived spousal support is related to PTG across time in HNC patients.

It is interesting to explore how common psychological complications related to cancer [such as depression, anxiety and posttraumatic stress symptoms (PTSSs)] are link to PTG among HNC patients. Overwhelming severity of depression, anxiety and PTSSs may disrupt the cognitive processing and search of meaning out of the trauma of living with cancer. Even if search for meaning out of the trauma of living with cancer occurred, if the search for meaning failed to solve unproductive ruminations such as cognitive posttraumatic stress symptoms, it may disrupt development of PTG (Ochoa Arnedo et al., 2019; Figure 1). Depression and anxiety are prevalent in patients with cancer because of the complications of facial disfigurement, which may increase the psychological vulnerability of patients because of society's emphasis on physical attractiveness (Long et al., 1996; Dropkin, 1999). The prevalence of depression and anxiety in HNC patients could be as high as 28 and 37%, respectively (Kugaya et al., 2000; Humphris et al., 2003; Massie, 2004). Therefore, evaluating whether depression and anxiety affect PTG in HNC patients would be interesting. Studies of PTG in other sites of cancer reported that there was only a weak negative association between depression and PTG, but there was an absence of association between anxiety and PTG (Shand et al., 2015). As for HNC patients, only a few studies have examined the relationship between depression, anxiety, and PTG. In a Malaysian study of HNC patients with a sample size of only 50 subjects, depression and anxiety were not found to be associated with PTG (Leong Abdullah et al., 2015). However, in a small-scale Dutch study of HNC survivors with psychological distress (*n* = 74), the absence of anxiety disorder was found to be associated with a higher degree of PTG (Holtmaat et al., 2017). To date, data are lacking on how depression and anxiety are related to PTG across time.

Besides depression and anxiety, PTSSs were reported in 33.4% of cancer survivors, and about 11.8% of survivors met the diagnostic criteria for posttraumatic stress disorder (PTSD) (Moschopoulou et al., 2018). Based on studies of PTG in patients diagnosed with cancer in other sites, PTSSs were weakly positively associated with PTG (Shand et al., 2015). Again, to the best of the authors' knowledge, no study has been conducted to date regarding how PTSSs are related to PTG over time. Therefore, the present longitudinal study was conducted to fill this research gap by (1) examining the trends of positive psychology (e.g., PTG, hope, and optimism), perceived spousal support, and psychological complications (e.g., depression, anxiety, and PTSSs) across time and (2) determining the association between positive psychology (e.g., hope and optimism), perceived spousal support, psychological complications (e.g., depression, anxiety, and PTSSs), and PTG across time after controlling for socio-demographic and clinical characteristics among a cohort of HNC patients.

## Methods

### Study Design and Respondent Recruitment

This longitudinal study was conducted from January 2019 to December 2020. The study population was recruited from HNC patients who were registered for treatment in two major tertiary referral centers for oncology in Malaysia. G^*^Power 3.1.9.7 was used for the calculation of sample size to compute two dependent means (matched pairs); with reference to a prospective study of HNC patients in Malaysia (Leong Abdullah et al., 2015), the effect size was 0.25, the α error was 0.05, and the power of the study was 0.8. Therefore, the sample size needed for the study was 152 subjects (after the inclusion of an estimated dropout rate of 20%). A total of 175 participants were drawn from a longitudinal study of the relationship between PTG and coping strategies in HNC patients (Nik Jaafar et al., 2021).

Consecutive sampling was used, in which HNC patients who attended the oncology and otorhinolaryngology units of the two targeted referral centers were approached by the research team and screened for eligibility criteria. The inclusion criteria were (1) those diagnosed with HNC, completed treatment within no more than 1 year, and at any stage of cancer (confirmed by a histopathological report); (2) those who are fit to answer questionnaires; and (3) those who were married or in a stable relationship for at least 6 months. The exclusion criteria were (1) those with a history of pre-existing mental illnesses (e.g., psychotic disorders, depressive disorders, bipolar mood disorder, anxiety disorders, and obsessive compulsive disorder), (2) those with a history of illicit drug and alcohol use, (3) those with a history of medical illnesses that may induce depressive and anxiety symptoms (e.g., hyperthyroidism, hypothyroidism, cerebrovascular accident, ischemic heart diseases, chronic obstructive airways disease, chronic pain, multiple sclerosis, Parkinson's disease, Addison's disease, Cushing's disease, and epilepsy), and (4) those with regular medications that may induce depressive and anxiety symptoms (e.g., clonidine, guanethidine, methyldopa, reserpine, beta blockers, isotretinoin, levetiracetam, triptans, corticosteroids, oral contraceptives, gonadotropin-releasing hormone agonists, tamoxifen, varenicline, interferons, and levodopa). Those who met all eligibility criteria were invited to participate in the study and signed informed consent forms before they were enrolled in the research. The purpose of the study, the study procedures, the participants' anonymity, and their right to withdraw from the study were explained to them. The research received approval from the Human Research Ethics Committee of the two targeted tertiary referral centers for oncology where the study subjects were recruited.

### Measures

Data collection was performed by a research assistant who was not involved in the study to avoid observer bias. Social desirability bias was minimized through the assurance of anonymity and confidentiality of the information that the respondents disclosed in this study. They were also not required to disclose any personal identification information during the assessments. Respondent assessments in this study were performed across two timelines, which were the baseline assessment (when the respondents were first enrolled in the study) and the follow-up assessment (which were between 5 and 7 months after the baseline assessment). During the baseline assessment, the respondents were administered the socio-demographic and clinical characteristics questionnaire, the Malay versions of the Posttraumatic Growth Inventory-Short Form (PTGI-SF), the Dispositional Hope Scale, the Sources of Social Support Scale (SSSS), the Life Orientation Test-Revised (LOT-R), the Hospital Anxiety and Depression Scale (HADS), and the PTSD Checklist for DSM-V (PCL-5). During the follow-up assessment, the respondents were re-administered with the question on the mode of treatment received, the Malay versions of the PTGI-SF, the Disproportional Hope Scale, the SSSS, the LOT-R, the HADS, and the PCL-5.

#### Outcome Variable (Posttraumatic Growth)

In this study, the Malay version of the PTGI-SF was used to measure the degree of PTG among the respondents. The PTGI-SF is a shorter version of the Posttraumatic Growth Inventory (PTGI), which could substitute for the PTGI without any loss of information. The PTGI-SF is a self-reporting instrument made up of 10 items designating five domains (appreciation of life, relating to others, spiritual development, personal strength, and new possibilities in life). Each item is scored on a Likert scale ranging from 0 to 5. Therefore, the total score may range from 0 to 50, with a higher score indicating a higher degree of PTG. The PTGI-SF exhibited good internal consistency with a Cronbach's α of 0.86 (Cann et al., 2010). The Malay version of the PTGI-SF has been validated in the Malaysian cancer population and reported good internal consistency with a Cronbach's α of 0.89; confirmatory factor analysis revealed that it has five domains similar to the original English version of the PTGI-SF (Leong Bin Abdullah et al., 2017a). In this study, the Cronbach's α of the Malay version of the PTGI-SF was 0.94, whereby the internal consistency was excellent.

#### Explanatory Variables

##### Hope

The Malay version of the Dispositional Hope Scale was administered to the respondents to assess their level of hope. It is a self-rated 12-item scale and consists of two subscales that incorporate Snyder's cognitive model of hope: (a) agency (goal-directed energy) and (b) pathways (planning to accomplish goals). Four of the 12 items assess agency, whereas another four items assess pathways. The other four items are fillers. Each item is scored using a Likert scale ranging from 1 (strongly disagree) to 4 (strongly agree) (Everson et al., 1996). Therefore, the total score ranges from 8 to 32. A higher score indicates a higher degree of hope. The Malay version of the Hope Scale was validated in Malaysian cancer patients and demonstrated acceptable internal consistency with a Cronbach's α of 0.716; confirmatory factor analysis revealed that it has two domains similar to the original English version of the Dispositional Hope Scale (Leong Bin Abdullah et al., 2018). In this study, the internal consistency of the Malay version of the Hope Scale registered a good Cronbach's α of 0.89.

##### Optimism

The Malay version of the LOT-R was administered to the respondents to assess their degree of optimism. The LOT-R is a self-rated tool derived from the Life Orientation Test, in which the LOT-R omitted or rewrote items that did not focus on explicit expectations. It consists of six items designated to two domains (optimism and pessimism). Three items are assigned to the optimism domain, and another three items are assigned to the pessimism domain. Each item is scored on a Likert scale ranging from 0 (strongly disagree) to 4 (strongly agree). A higher score indicates a higher degree of optimism. It has good internal consistency and is stable over time. The positive and negative subsets are more strongly related to each other than those in LOT (Scheier et al., 1994). The Malay version of the LOT-R was validated in the Malaysian cancer population and exhibited a Cronbach's α of 0.58. Confirmatory factor analysis revealed that it has two domains similar to the original English version of the LOT-R (Leong Bin Abdullah et al., 2017b). In this study, the internal consistency of the Malay version of the LOT-R exhibited a Cronbach's α of 0.57.

##### Perceived Spousal Support

The Malay version of the SSSS was used to assess the degree of perceived spousal support among the respondents in this study. The SSSS is a self-rated questionnaire consisting of 10 items scored on a Likert scale ranging from 1 = not at all to 5 = a lot. Therefore, the total score ranges from 10 to 50. A higher score denotes a greater degree of perceived spousal support (Kinsinger et al., 2011). The Malay version of the SSSS was validated in Malaysian cancer patients and exhibited acceptable internal consistency with a Cronbach's α of 0.72 (Leong Bin Abdullah et al., 2017c). In this study, the internal consistency of the Malay version of the SSSS was acceptable with a Cronbach's α of 0.74.

##### Depression and Anxiety

The Malay version of HADS was used to measure the degree of depressive and anxiety symptoms experienced by the respondents. HADS is a self-rated questionnaire with seven items designated to the depression subscale and another seven items designated to the anxiety subscale. It is suitable for assessing the severity of depression and anxiety symptoms in patients with medical illnesses, such as cancer patients, as HADS focuses on psychological symptoms rather than physical symptoms; the latter, as well as depression and anxiety, may be present in cancer. Each item is scored from 0 to 3, and the range of total scores for both depressive and anxiety subscales is 0 to 21 per subscale (Zigmond and Snaith, 1983). The cut-off for caseness of depression is 8/21, and that for caseness of anxiety is also 8/21. The anxiety subscale has a sensitivity of 0.9 and a specificity of 0.78, and the depression subscale has a sensitivity of 0.83 and a specificity of 0.79 (Bjelland et al., 2002). The Malay version of HADS was validated in Malaysian breast cancer patients and exhibited acceptable to good internal consistency for its total score and subscales, with a Cronbach's α ranging from 0.73 to 0.87 (Yong et al., 2016). In this study, the Malay version of HADS reported an excellent internal consistency with a Cronbach's α of 0.90.

##### Posttraumatic Stress Disorder Symptoms

The Malay version of the PCL-5 was administered to the respondents to evaluate their degree of PTSSs. The PCL-5 is a self-rated tool that consists of 20 items derived from the Diagnostic and Statistical Manual for Mental Disorders diagnostic criteria for PTSD. Each item is scored in a Likert scale ranging from 0 = “Not at all” to 4 = “Extremely.” Therefore, its total score may range from 0 to 80. The PCL-5 exhibits good psychometric properties (Blevins et al., 2015). The Malay version of the PCL-5 was validated in the Malaysian population that had traumatic experiences, and it showed good psychometric properties with strong internal consistency (Cronbach's α = 0.89; Bahari et al., 2019). In this study, the internal consistency of the Malay version of the PCL-5 was excellent with a Cronbach's α of 0.96.

#### Sociodemographic and Clinical Characteristics

The data collected on the sociodemographic characteristics of the respondents included gender, age, monthly household income, and education. The responses to the question on gender were documented as either “male” or “female.” The responses to the item on age were recorded as “18 to 40 years old,” “41 to 60 years old,” and “more than 60 years old.” The responses to monthly household income were documented as “less than RM 3000,” “RM 3000 to RM 6000,” and “more than RM 6000.” Finally, the responses to education were recorded as “up to primary education,” “up to secondary education,” and “up to tertiary education.”

Regarding clinical characteristics, data were collected on the following: type of HNC, time since treatment completion, stage of cancer, and modalities of treatment received and completed. The responses to the item on type of HNC were documented as “nasopharyngeal carcinoma,” “oral cancer,” “thyroid cancer,” and “other types of head and neck cancers.” The responses to the time since treatment completion were reported as either “less than 6 months” or “6 to 12 months.” The responses to the item on stage of cancer were documented as “stage 1,” “stage 2,” “stage 3,” and “stage 4.” Finally, the responses to the item on the modalities of treatment received and completed were recorded as “surgery only,” “chemotherapy only,” “surgery and chemotherapy,” “surgery and radiotherapy,” “chemotherapy and radiotherapy,” and “surgery, chemotherapy and radiotherapy.”

### Statistical Analysis

Data analysis was performed with the Statistical Package for Social Sciences version 26 (SPSS 26). Descriptive statistics were reported for socio-demographic and clinical characteristics, the total PTGI-SF, total HS, total LOT-R, HADS depressive and anxiety subscale, total PCL-5, and total SSSS scores at baseline and follow-up assessments. The categorical variables were presented as frequencies and percentages. The continuous variables were described as means and standard deviations, as the Shapiro-Wilk test indicated that the continuous variables were normally distributed (*p* > 0.05). There were no missing data.

To achieve objective (1) of the study, a paired *t*-test was applied to compare the differences in the total PTGI-SF, total HS, total LOT-R, HADS depressive and anxiety subscale, total PCL-5, and total SSSS scores between baseline and follow-up assessments. To achieve objective (2) of the study, a random intercept model was applied, as the measures were not conducted on a predetermined fixed schedule (the time interval between baseline and follow-up assessments ranged from 5 to 7 months). Statistical significance was set at *p* < 0.05 for the paired *t*-test and repeated measures mixed effect model, and it was two sided.

As gender was a significant factor associated with PTG in cancer patients (Shand et al., 2015), a subgroup analysis was performed to evaluate the moderating effect of gender on the association between significant factors identified in the random intercept model and PTG by moderator analysis with a dichotomous moderator (gender). Statistical significance was set at *p* < 0.05 and it was two-sided.

## Results

### Respondents' Characteristics

The sociodemographic and clinical characteristics of the respondents are summarized in Table 1. Males constituted slightly more than half of the total respondents, and seven-tenths of the respondents were in the middle age group. Half of the respondents were diagnosed with nasopharyngeal carcinoma and had completed treatment within <6 months. More than half of the respondents were at stage 2 and stage 3 of cancer. Majority of the respondents received and completed at least two modalities of cancer treatment.

**Table 1:** Socio-demographic and clinical characteristics of respondents.

**Variables**	**Frequency (*n*)**	**Percentage (%)**
**Gender**		
Male	94	53.7
Female	81	46.3
**Age**		
18–40 years	10	5.7
41–60 years	125	71.4
>60 years	40	22.9
**Monthly household income**		
< RM 3,000	132	75.4
RM 3,000–6,000	28	16
>RM 6,000	15	8.6
**Education**		
Primary education	45	25.7
Secondary education	73	41.7
Tertiary education	57	32.6
**Types of head and neck cancer**		
Nasopharyngeal carcinoma	89	50.9
Oral cancer	40	22.9
Thyroid cancer	25	14.3
Others	21	12
**Time since completion of treatment**		
<6 months	89	50.9
6–12 months	86	49.1
**Stage of cancer**		
Stage 1	38	21.7
Stage 2	51	29.1
Stage 3	53	30.3
Stage 4	33	18.9
**Treatment modalities received and completed**		
Surgery only	7	5.7
Chemotherapy only	24	13.7
Surgery and chemotherapy	23	13.1
Surgery and radiotherapy	23	13.1
Chemotherapy and radiotherapy	54	30.9
Chemotherapy, radiotherapy,	44	25.1
and surgery		

### Trend of Posttraumatic Growth, Positive Psychology, and Psychological Complications Across Time

The mean total PTGI-SF, SSSS, LOT-R, Dispositional Hope Scale, PCL-5, and HADS subscale scores during the baseline and follow-up assessments are shown in Table 2. The degree of positive psychology, such as PTG, hope, optimism, and perceived spousal support, exhibited a significantly increasing trend from baseline to follow-up. Conversely, the degree of psychological complications, such as depressive symptoms, anxiety symptoms, and PTSSs, showed a significantly decreasing trend from baseline to follow-up.

**Table 2:** Mean total PTGI-SF, SSSS, LOT-R, Dispositional Hope Scale, PCL-5, and HADS subscale scores during baseline and follow up assessments.

**Variables**	**Baseline assessment**		**Follow up assessment**		***p*-value**
	**Mean**	**Standard deviation**	**Mean**	**Standard deviation**	
Total PTGI-SF score	34.05	11.20	39.43	9.40	<0.001^*^
Total SSSS score	40.62	6.78	42.11	6.47	0.023^*^
Total LOT-R score	13.83	2.44	14.86	3.37	0.002^*^
Total Hope Scale score	24.87	3.84	26.97	2.21	<0.001^*^
HADS anxiety subscale	6.95	4.23	5.41	4.54	<0.001^*^
HADS depression subscale	6.63	4.37	5.25	4.30	0.002^*^
Total PCL-5 score	15.5	14.57	12.87	14.28	0.047^*^

* *Statistical significance at p <0.05*;

*PTGI-SF, Posttraumatic Growth Inventory-Short Form; SSSS, Source of Social Support Scale; LOT-R, Life Orientation Test-Revised; HADS, Hospital Anxiety and Depression Scale; PCL-5, PTSD checklist for DSM-V*.

### Association Between Positive Psychology, Psychological Complications, Sociodemographic and Clinical Characteristics, and PTG Across Time

The associations between the total SSSS, LOT-R, Dispositional Hope Scale, PCL-5, and HADS subscale scores, sociodemographic and clinical characteristics, and the total PTGI-SF score across the timeline between baseline and follow-up assessments are presented in Table 3. The random intercept model revealed that the only positive psychology significantly associated with PTG across time was hope. A greater degree of hope significantly contributed to a higher degree of PTG among the respondents [estimate = 0.098, 95% confidence interval (CI) = 0.003–0.193, standard error (SE) = 0.048, *t* = 2.020, *p* = 0.044]. In addition, a greater degree of perceived spousal support was significantly associated with a higher degree of PTG across time (estimate = 0.515, 95% CI = 0.349–0.681, SE = 0.084, *t* = 6.098, *p* < 0.001). Contrastingly, anxiety symptoms were the only psychological complications significantly associated with PTG across time, in which a higher degree of anxiety symptoms contributed to a lower level of PTG among the respondents (estimate = −0.529, 95% CI = −0.922 to −0.136, SE = 0.200, *t* = −2.650, *p* = 0.008).

**Table 3:** The random intercept model between the socio-demographic and clinical characteristics, total SSSS, total Hope Scale, total LOT-R, total PCL-5 scores, HADS subscale scores (independent variables), and total PTGI-SF scores (dependent variable).

**Variables**	**Estimate (95% CI)**	**Standard error**	***t***	***p*-value**
**Gender**				
Female	Reference			
Male	−2.882 (−5.179 to −0.583)	1.164	−2.474	0.014^*^
**Age**				
18–40 years	Reference			
41–60 years	2.926 (−3.971 to 7.823)	2.989	0.645	0.52
>60 years	0.663 (−4.669 to 6.000)	2.702	0.246	0.806
**Monthly household income:**
< RM 3,000	Reference			
RM 3,000–6,000	−2.347 (−6.852 to 2.159)	2.283	−1.028	0.305
>RM 6,000	−0.767 (−5.517 to 3.983)	2.407	−0.319	0.75
**Education**				
Primary education	Reference			
Secondary education	2.391 (−1.097 to 5.879)	1.767	1.353	0.178
Tertiary education	−0.681 (−3.643 to 2.282)	1.501	−0.454	0.651
**Types of head and neck cancer**				
Others	Reference			
Nasopharyngeal carcinoma	−0.492 (−3.576 to 2.591)	1.563	−0.35	0.753
Oral cancer	−1.027 (−4.751 to 2.697)	1.887	−0.544	0.587
Thyroid cancer	−0.060 (−4.585 to 4.464)	2.293	−0.026	0.979
**Time since completion of treatment**				
<6 months	Reference			
6–12 months	−0.361 (−2.619 to 1.898)	1.145	−0.315	0.753
**Stage of cancer**				
Stage 1	Reference			
Stage 2	0.941 (−2.287 to 4.170)	1.636	0.576	0.566
Stage 3	0.948 (−2.452 to 4.349)	1.723	0.551	0.583
Stage 4	2.308 (−1.417 to 6.032)	1.887	1.223	0.223
**Treatment modalities received and completed**				
Surgery only	Reference			
Chemotherapy only	3.208 (−2.535 to 8.951)	2.91	1.103	0.272
Surgery and chemotherapy	0.144 (−5.615 to 5.903)	2.918	0.049	0.961
Surgery and radiotherapy	0.421 (−5.403 to 6.244)	2.951	0.143	0.887
Chemotherapy and radiotherapy	0.992 (−4.512 to 6.495)	2.788	0.356	0.723
Chemotherapy, radiotherapy, and surgery	−0.850 (−6.233 to 4.533)	2.727	−0.312	0.756
**Total SSSS score**	0.515 (0.349 to 0.681)	0.084	6.098	<0.001^*^
**Total LOT-R score**	0.215 (−0.166 to 0.595)	0.194	1.108	0.269
**Total Hope Scale score**	0.098 (0.003 to 0.193)	0.048	2.02	0.044^*^
**HADS anxiety subscale**	−0.529 (−0.922 to −0.136)	0.2	−2.650	0.008^*^
**HADS depression subscale**	0.086 (−0.296 to 0.468)	0.194	0.442	0.659
**Total PCL-5 score**	0.125 (-0.030 to 0.280)	0.079	1.584	0.114

* *Statistical significance at p < 0.05*.

As for sociodemographic and clinical characteristics, the random intercept model indicated that gender was the only factor significantly associated with PTG across time. Males exhibited a significantly lower level of PTG than females across time (estimate = −2.882, 95% CI = −5.179 to −0.583, SE = 1.164, *t* = −2.474, *p* = 0.014).

### Moderating Effect of Gender in the Association Between Hope, Perceived Spousal Support, Severity of Anxiety Symptoms, and PTG

The moderating effect of gender on the association between hope and PTG among the HNC respondents are summarized in Table 4. In model 1, gender (*p* = 0.029) and hope (*p* = 0.015) were significantly associated with PTG. The linear regression model contributed to a significant regression equation of F_(2,172)_ = 3.721 with *R*^2^ = 0.041 and *p* = 0.026. In model 2, when the interaction between female and hope was added to the model, the *R*^2^ change (0.004) was non-significant (*p* = 0.591), indicating that the female gender did not moderate the association between hope and PTG.

**Table 4:** The moderating effect of gender on the association between the total Hope Scale score and total PTGI-SF score.

**Variables**	**B (95% CI)**	**Standard error**	***t***	***p*-value**
**(Model 1)^a^**				
**Gender**				
Male	Reference			
Female	2.020 (1.281–5.321)	1.672	1.208	0.029^*^
Total Hope Scale	0.536 (0.106–0.965)	0.218	2.463	0.015^*^
**(Model 2)^b^**				
**Gender**				
Male	Reference			
Female	1.986 (−0.085 to 3.657)	1.587	1.190	0.177
**Total Hope Scale**	0.565 (−0.114 to 1.243)	0.641	1.656	0.102
**Female x Hope Scale**	0.301 (−0.810 to 1.411)	0.558	0.539	0.591

* *Statistical significance at p < 0.05*.

a *F_(2,172)_ = 3.721, R^2^ = 0.041, p = 0.026*.

b *R^2^ change = 0.004, p = 0.591*.

The moderating effect of gender on the association between perceived spousal support and PTG among the HNC respondents are presented in Table 5. In model 1, gender (*p* = 0.043) and perceived spousal support (*p* < 0.001) were significantly associated with PTG. The linear regression model contributed to a significant regression equation of F_(2,172)_ = 16.417 with *R*^2^ = 0.160 and *p* < 0.001. In model 2, when the interaction between female and perceived spousal support was added to the model, the *R*^2^ change (0.025) was non-significant (*p* = 0.150), depicting that the female gender did not moderate the association between perceived spousal support and PTG.

**Table 5:** The moderating effect of gender on the association between the total SSSS score and total PTGI-SF score.

**Variables**	**B (95% CI)**	**Standard error**	***t***	***p*-value**
**(Model 1)^a^**				
**Gender**				
Male	Reference			
Female	2.670 (0.079–5.261)	1.313	2.034	0.043^*^
Total SSSS	0.543 (0.343–0.744)	0.102	5.352	<0.001^*^
**(Model 2)^b^**				
**Gender**				
Male	Reference			
Female	1.967 (−0.090 to 3.234)	1.456	1.090	0.145
**Total SSSS**	0.320 (−0.007 to 0.647)	0.164	1.951	0.055
**Female x SSSS**	0.250 (−0.093 to 0.593)	0.172	1.454	0.150

* *Statistical significance at p < 0.05*.

a *F_(2,172)_ = 16.417, R^2^ = 0.160, p < 0.001*.

b *R^2^ change = 0.025, p = 0.150*.

Finally, the moderating effect of gender on the association between severity of anxiety symptoms and PTG among the HNC respondents are illustrated in Table 6. In model 1, gender (*p* = 0.048) and severity of anxiety symptoms (*p* = 0.001) were significantly associated with PTG. The linear regression model contributed to a significant regression equation of F_(2,172)_ = 7.143 with *R*^2^ = 0.077 and *p* = 0.001. In model 2, when the interaction between female and severity of anxiety symptoms was added to the model, the *R*^2^ change (0.065) was significant (*p* = 0.022), denoting that the female gender moderated the association between severity of anxiety symptoms and PTG. Higher interaction between female gender and severity of anxiety symptoms contributed to significantly higher degree of PTG (B = 0.625, 95% CI = 0.091–1.160, SE = 0.269, *t* = 2.328, *p* = 0.022).

**Table 6:** The moderating effect of gender on the association between the HADS anxiety subscale score and total PTGI-SF score.

**Variables**	**B (95% CI)**	**Standard error**	***t***	***p*-value**
**(Model 1)^a^**				
**Gender**				
Male	Reference			
Female	2.743 (0.026–5.460)	1.377	1.993	0.048^*^
HADS anxiety subscale	−0.490 (−0.790 to −0.191)	0.152	−3.237	0.001^*^
**(Model 2)^b^**				
**Gender**				
Male	Reference			
Female	2.867 (0.054–5.670)	1.567	2.345	0.034^*^
**HADS anxiety subscale**	−0.045 (−0.674 to 0.583)	0.316	−0.144	0.886
**Female** **×HADS anxiety subscale**	0.625 (0.091–1.160)	0.269	2.328	0.022^*^

* *Statistical significance at p <0.05*.

a *F_(2,172)_ = 7.143, R^2^ = 0.077, p = 0.001*.

b *R^2^ change = 0.065, p = 0.022*.

## Discussion

This longitudinal study assessed the trend of positive psychology (e.g., PTG, hope, and optimism), perceived spousal support, and psychological complications (e.g., depressive, anxiety, and PTSSs) and determined the association between positive psychology, perceived spousal support, psychological complications, and PTG across two timeframes in a cohort of HNC patients who had completed their cancer treatment within no more than 1 year. Our findings revealed that the degree of positive psychology (e.g., PTG, hope, and optimism) and perceived spousal support increased across time, whereas psychological complications (e.g., depressive, anxiety, and PTSSs) decreased across time. A higher degree of hope and perceived spousal support contributed to a higher level of PTG, whereas a greater severity of anxiety symptoms lowered the level of PTG across time among the HNC respondents. Nevertheless, optimism, depression, and PTSSs were not associated with PTG over time.

Studies have reported that positive psychology exhibits an increasing trend across time in cancer patients who are recently diagnosed. PTG demonstrated an increasing trend during the first 18 months after HNC treatment completion before it plateaued off after 18 months (Harding, 2018). The findings in this study confirmed the trend of PTG across time, as the HNC respondents were within the first 18 months after the completion of their cancer treatment. Regarding the trend of other forms of positive psychology across time, the degree of hope among cancer patients treated with curative intent increased over time compared with those who were treated with palliative intent (Sanatani et al., 2008). Similarly, the degree of optimism among newly diagnosed HNC patients also showed an increasing trend over time and was reported to predict 1-year survival (Allison et al., 2003). Our study sample, which consisted of individuals all treated with curative intent and completed cancer treatment within no more than 1 year, also exhibited similar increasing trends of hope and optimism across time, shedding new light on how the degree of hope and optimism varies over time.

As for perceived spousal support, a study by Song et al. (2012) among 134 couples (prostate cancer patients and their spouses) reported a significant increase in perceived spousal support and a significant decrease in uncertainty among cancer patients between baseline assessment and follow-up assessment after 12 months. The increasing trend of perceived spousal support over time was contributed by increased perceived communication between cancer patients and their spouses (Song et al., 2012). Therefore, the increasing trend of perceived spousal support among the HNC respondents in this study may be attributed to the increased communication between the respondents and their spouses after the diagnosis of cancer.

The prevalence of depression increased 3 weeks after the completion of cancer treatment, but it decreased 18 months after treatment completion in newly diagnosed HNC patients (Neilson et al., 2013). Our study findings supported the trend of depression in HNC patients, as depression decreased ~18 months after the completion of cancer treatment (during the follow-up assessment). Regarding anxiety in HNC patients, its prevalence was high during pre-treatment, but it decreased over time after treatment completion (Wu et al., 2016). Our findings further confirmed the trend of anxiety over time in HNC patients, as reported in other studies in the HNC population. As for the trend of PTSSs in patients diagnosed with cancer in various sites, the prevalence of PTSSs was high at 3–6 months after diagnosis and completion of treatment, but it followed a decreasing trend thereafter (O'Connor et al., 2011; Chan et al., 2018). This finding is in line with those reported in studies of patients with different sites of cancer, highlighting that the trend of PTSSs over time may be universal across patients with different sites of cancer.

Our study findings indicated that a higher degree of hope contributed to a higher degree of PTG among HNC patients over time, whereas optimism was not associated with PTG. In addition, a meta-analysis of studies of PTG in cancer patients revealed that optimism is only weakly correlated with PTG (Shand et al., 2015). Similar findings were also reported by Ho et al. (2011) in a study of oral cancer patients. It has been suggested that hope is a component of meaning making. Therefore, a higher degree of hope would allow cognitive reprocessing of trauma-related information of living with cancer and the adverse effects of its treatment, which may facilitate the search for meaning out of the trauma and facilitate the development of PTG (Hullmann et al., 2014). A higher degree of hope is also positively related to greater perceived social support, in which cancer patients with more hope may tend to utilize their social networks in order to cope with their highly stressful experience of living with cancer, thus enhancing the interpersonal relationship between the patients and their social network. This facilitates the development of PTG. A higher degree of hope is also associated with cognitive flexibility, in which cancer patients successfully readjust their goals in relation to the new circumstances of living with cancer, which may increase new possibilities in life. Moreover, a greater degree of hope is associated with higher self-efficacy. This may enhance patients' personal strengths and, in turn, increase their degree of PTG. Cancer patients with a higher level of hope may have a higher probability of finding meaning and benefitting out of the highly stressful experience of living with cancer, which may allow greater appreciation of life and facilitate the development of PTG (Leong Abdullah et al., 2019). Unlike hope, optimism is related only to PTG in certain circumstances, such as in cancer survivors who perceive that they have better control over their stress (Leong Abdullah et al., 2019).

In a cross-sectional study, a higher degree of social support was well-documented to predict a higher degree of PTG among HNC patients (Sharp et al., 2018). A meta-analysis of studies on PTG in cancer survivors also highlighted that increasing social support has a moderate effect on enhancing PTG in cancer survivors (Prati and Pietrantoni, 2009; Shand et al., 2015). The present study confirmed that social support, especially spousal support, could indeed contribute to facilitating the development of PTG in HNC survivors. Research has shown that just talking and venting any emotional turmoil that cancer patients experience to close family members could enhance emotional support from family members. This self-disclosure of thoughts and emotions could enhance personal strength in cancer patients, which, in turn, facilitates the development of PTG (Leong Abdullah et al., 2019). A higher degree of spousal support could also facilitate cognitive reprocessing of the trauma-related event of living with cancer, which, in turn, initiates meaning making and eventually facilitates the development of PTG (Schroevers et al., 2010).

On the contrary, a greater severity of anxiety symptoms was the only psychological complication associated with lower PTG across time. Our study confirmed the finding of a cross-study of PTG in Dutch HNC survivors, which reported that the presence of anxiety disorder, but not depression, lowers PTG among the survivors (Holtmaat et al., 2017). Similarly, a negative association between anxiety and PTG was also reported in breast cancer patients in the first year after the completion of cancer treatment (Wang et al., 2014; Canavarro et al., 2015). The HNC survivors in our study may be heavily burdened by anxiety symptoms that they failed to experience cognitive processing and search for meaning out of the traumatic event of living with cancer (Holtmaat et al., 2017). Hence, this may disrupt the development of PTG resulting in lowering of PTG.

Similarly, our findings indicated that PTSSs was not associated with PTG across time in HNC survivors. PTSSs exhibited a curvilinear relationship with PTG in cancer patients, in which PTSSs at milder severity increased PTG but as its severity increase further, PTG reduces (Shand et al., 2015). Again, overwhelming psychological distress may dissipate the cognitive processing and search for meaning out of the trauma of living with cancer and lead to failure to develop PTG (Zhang et al., 2022), as depicted in our finding.

In the context of the moderating effect of gender on the association between hope, perceived spousal support, severity of anxiety symptoms, and PTG; the only significant moderating effect of female gender was on the association between severity of anxiety symptoms and PTG. Surprisingly, the interaction between female gender and severity of anxiety symptoms contributed to higher degree of PTG. Female cancer patients tend to cope with emotional coping and may have a higher tendency to perceive living with cancer as traumatic (Tanyi et al., 2017; Sharp et al., 2018). Hence, this increases the possibility of developing PTG even in the presence of anxiety symptoms related to living with cancer. In essence, despite having anxiety symptoms, female may still be having higher probability of developing PTG compared to male HNC patients as denoted by our study findings.

Our findings should be interpreted in consideration of a few limitations. First, the socio-demographic and clinical characteristics of the respondents in this study may not be fully representative of the HNC patient population in Malaysia. This may affect the generalizability of our findings. Second, there were only two time points of assessments in this study, which may not be sufficient to evaluate how the relationship between the associated factors and PTG varies with time. Finally, this study did not assess the physical symptoms of HNC and adverse effects of its treatment which could affect the health-related quality of life of patients and the development of PTG (such as problem with chewing, swallowing, tasting of food, thick saliva, restriction of physical and recreational activities) during the time interval between baseline and follow up assessments (Harding, 2018). This may be an important confounding factor that may affect the findings of the study.

Despite these limitations, to date, this was the first longitudinal study to evaluate the association between positive psychology (e.g., hope and optimism) and PTG across time in HNC patients while controlling for psychological complications (e.g., depression, anxiety, and PTSSs). The relationship between another important associated factor, perceived spousal support, and PTG across time was also measured in this longitudinal study. Data on how these factors were related to PTG across time in HNC patients were lacking. Our study provided valuable data on psychosocial factors associated with increase or decrease in PTG across time, so that psychosocial interventions which facilitate associated factors that increase PTG and mitigate associated factors that decrease PTG could be formulated to safeguard the mental wellbeing of HNC patients and incorporated as part of the treatment regimen for HNC.

In essence, it should be noted that not all HNC patients living with cancer will develop PTG. If the traumatic experience of living with cancer is too intense, it may disrupt the cognitive reprocessing of the traumatic event to search for meaning, hence reducing the likelihood of PTG occurrence (Zhang et al., 2022). Hence, psychosocial interventions which enhance the development of PTG are needed as part of the treatment regime for HNC patients. Based on our study findings, we recommend that treating clinicians include psychosocial interventions that may enhance spousal support and caretaker support for cancer patients, such as social skills training, psychoeducation, and therapeutic counseling (Northouse et al., 2010). Enhancing spousal support will increase the probability of developing PTG and improve the wellbeing of HNC patients. In addition, hope enhancement strategies may promote the development of PTG among HNC patients and should be incorporated into the treatment regime by treating clinicians. It is also advisable to screen for the severity of anxiety symptoms among HNC patients and ensure that anxiety is thoroughly managed with psychosocial interventions (e.g., cognitive behavioral therapy, mindfulness-based interventions, or acceptance and commitment therapy) to facilitate the development of PTG and improvement in the mental wellbeing of patients.

## Conclusion

This longitudinal study showed that the degree of positive psychology (e.g., PTG, optimism, and hope) exhibited an increasing trend, whereas psychological complications (e.g., depression, anxiety, and PTSSs) showed a decreasing trend across a timeline of between 5 and 7 months after treatment completion among HNC patients. A higher degree of spousal support and hope contributed to a higher degree of PTG across time, but a higher severity of anxiety symptoms decreased the degree of PTG over time. Conversely, optimism, depression, and PTSSs did not contribute to changes in PTG across time. This study provided useful data for treating clinicians on the pivotal roles of screening for the degree of hope, perceived spousal support, and severity of anxiety symptoms among HNC patients and of incorporating psychosocial interventions to manage these variables in order to facilitate development of PTG and improvements in the mental wellbeing of patients.

## Data Availability Statement

The raw data supporting the conclusions of this article will be made available by the authors, without undue reservation.

## Ethics Statement

The studies involving human participants were reviewed and approved by the Human Research Ethics Committee of Universiti Sains Malaysia; the Human Research Ethics of Faculty of Medicine, Universiti Kebangsaan Malaysia. The patients/participants provided their written informed consent to participate in this study.

## Author Contributions

ML and NS conceptualized and design the study. ML, NS, NN, NA, NM, RH, SI, NH, RR, RM, MM, and HZ involved in data collection. ML, NS, and NN involved in data and statistical analysis. ML wrote the first draft of the manuscript. All authors involved in the revision of the manuscript and approved the submitted version.

## Funding

This work was supported by the Short Term Grant of Universiti Sains Malaysia (Grant Number: 304/CIPPT/6315236) (ML). The funder has no role in the conceptualization of the review, literature review, writing of the manuscript, and decision on submission of the manuscript for publication. There was no fund received for open access publication fees.

## Conflict of Interest

The authors declare that the research was conducted in the absence of any commercial or financial relationships that could be construed as a potential conflict of interest.

## Publisher's Note

All claims expressed in this article are solely those of the authors and do not necessarily represent those of their affiliated organizations, or those of the publisher, the editors and the reviewers. Any product that may be evaluated in this article, or claim that may be made by its manufacturer, is not guaranteed or endorsed by the publisher.
